# Association Between Olympic Basketball Participation and Subsequent NBA Season Performance and Injury Incidence

**DOI:** 10.1177/23259671261449154

**Published:** 2026-06-18

**Authors:** Vineet Kumar, Luke Sang, Wyatt David, Nirav Pandya

**Affiliations:** * Department of Orthopaedic Surgery, University of California San Francisco, San Francisco, California, USA; † School of Medicine, University of California San Francisco, San Francisco, California, USA; Investigation performed at the Department of Orthopaedic Surgery, University of California, San Francisco, California, USA

**Keywords:** Olympic participation, injury incidence, NBA performance, workload management

## Abstract

**Background:**

While Olympic participation offers elite basketball players international competition, it occurs shortly after a demanding National Basketball Association (NBA) season, limiting recovery time. Condensed scheduling and cumulative physical stress may elevate injury risk in the subsequent professional season. Understanding how Olympic involvement affects player health and performance is essential for optimizing player sustainability within the NBA.

**Hypothesis:**

NBA players competing in the Summer Olympics will demonstrate higher injury incidence and reduced durability in the subsequent season, without improvement in performance metrics.

**Study Design:**

Descriptive epidemiology study.

**Methods:**

Publicly available NBA player data from the 2003-2004 to 2024-2025 seasons were reviewed. Inclusion was limited to NBA players who represented the United States in the Olympic Games with documented participation in the NBA season both immediately preceding and immediately following their Olympic appearance. A total of 67 players were included. Variables analyzed included regular season metrics (age, years of NBA experience, number of games played, total minutes played, plus-minus, net rating, game score), injury incidence during the regular season and postseason, and Olympic workload, defined as total minutes played during the Olympic tournament. Data were adjusted for shortened seasons (2011-2012, 2020-2021). Paired *t* tests and multivariable linear regression were performed.

**Results:**

The percentage of games played declined significantly in the NBA season following Olympic participation compared with the preceding NBA season (89.2% ± 10.3% vs 83.2% ± 18.7%; *P* = .01; *d* = −0.31). Injury incidence increased in the subsequent NBA season compared with the preceding season (0.7 ± 0.8 vs 0.9 ± 1.0; *P* = .04; *d* = 0.25). No significant differences were observed in plus-minus (*P* = .44; *d* = 0.096), net rating (*P* = .65; *d* = 0.055), game score (*P* = .49; *d* = 0.086), or total minutes played (*P* = .55; *d* = −0.07).

**Conclusion:**

Olympic participation was associated with increased injury incidence and decreased availability in the subsequent NBA season, without meaningful changes in performance metrics or total minutes played. These findings suggest cumulative fatigue and limited offseason recovery may be associated with overuse-related injury and reduced durability, with age and preceding injury history as additional risk factors.

The Summer Olympics occur every 4 years, with top basketball players from professional leagues such as the National Basketball Association (NBA) competing in an intensive tournament.^27^ Participating in the Olympics is considered a great honor, as it allows players to represent their native countries and gain significant publicity. However, playing in the Olympics after a prolonged regular season and playoffs introduces notable physical challenges related to cumulative workload and shortened recovery intervals, which may adversely affect player health and subsequent performance.^12,22^

The NBA regular season consists of 82 games, with playoff participation potentially adding up to 28 additional games. Despite this schedule, Olympic training camps often begin within weeks of the NBA finals, thereby limiting the time available for initial recovery. The Olympic tournament extends through group and elimination play, frequently concluding less than a month before NBA training camp begins, further compressing the offseason recovery period.

During a regular offseason, players rely on this time for rest, training, or even rehabilitation from injuries sustained during the 9-month season.^18^ When rest is shortened by Olympic participation, athletes may be more susceptible to cumulative fatigue and delayed recovery, which could adversely affect readiness and performance in the subsequent season, as has been observed in other sports with condensed competitive schedules.^10,13,14,15,20,23,26^ The risk of cumulative fatigue and impaired recovery is supported by prior NBA workload literature demonstrating that increased performance load and cumulative fatigue are associated with higher injury risk and greater injury incidence as the season progresses.^2,5,7,20,24^ Additionally, schedule-related factors such as calendar congestion and back-to-back games have been associated with increased fatigue, incomplete recovery, and impaired performance, further highlighting the importance of adequate rest intervals.^11,17,29,30^ These findings align with the growing emphasis on workload management in the NBA, where players may utilize in-season rest strategies to compensate for anticipated reductions in offseason recovery associated with Olympic participation. Nonetheless, such adaptations may not fully mitigate cumulative fatigue and may carry downstream consequences for player availability and injury risk. Previous studies in other sports, including both hockey and soccer, have shown that condensed schedules are associated with higher injury incidence and reduced performance, raising concern that Olympic participation could have similar effects among elite basketball players.^1,3,9^

Despite these recognized risks, there are potential benefits to competing in the Olympics, including better conditioning and exposure to diverse playing environments that can prepare players for better NBA performance. However, the potential adverse effects of Olympic participation on player health and performance have not been fully explored within the NBA. Therefore, understanding the relationship between Olympic participation and subsequent NBA performance and injury risk is crucial for maximizing player performance and protecting long-term health. From a clinical and sports medicine perspective, these insights are essential for informing return-to-play decisions, optimizing workload strategies, and reducing injury risk in elite athletes exposed to condensed competitive schedules. This study aims to determine whether participation in the Olympic Games is associated with subsequent professional basketball performance and injury rates. We hypothesize that players who compete in the Olympics will demonstrate a higher incidence of injury and reduced availability in the subsequent season, without significant changes in performance metrics compared with the season preceding Olympic participation.

## Methods

A retrospective cohort study was performed analyzing NBA player performance and injury data before and after participation in the Summer Olympics. Data were accessed from publicly available sources, including the NBA.com advanced statistics database and Basketball-Reference, for NBA players from the 2003-2004, 2004-2005, 2007-2008, 2008-2009, 2011-2012, 2012-2013, 2015-2016, 2016-2017, 2019-2020, 2020-2021, 2023-2024, and 2024-2025 seasons. Players who participated in the 2004, 2008, 2012, 2016, 2020, or 2024 Olympic Games were identified. As this study utilized publicly available data, it was deemed exempt from institutional review board approval. Inclusion criteria required that NBA players represent the United States in the Olympic Games and compete in both the preceding and the subsequent NBA seasons. Exclusion criteria included players who did not participate in an NBA season both preceding and following the Olympic Games; players with insufficient game participation due to non–injury related absences (eg, repeated “did not play − coach’s decision”), defined as missing ≥20 games for non–injury related reasons; and players with injuries sustained prior to the preceding NBA season that limited performance data availability. International players representing non-US national teams were also excluded because of differences in workload and playing time that could introduce variability in performance and injury outcomes.

Variables analyzed included age and years of NBA experience at the time of Olympic participation. Pre-Olympic NBA regular season variables included total games played, total minutes played, plus-minus (team point differential while the player is on the court), game score (a composite measure of individual player performance), net rating (team point differential per 100 possessions while the player is on the court), number of injuries, and injury severity (defined by games missed). Post-Olympic NBA regular season variables included total games played, total minutes played, plus-minus, game score, net rating, number of injuries, and injury severity (defined by games missed). Olympic workload was defined as total minutes played during the Olympic tournament. Pre- and post-Olympic season variables were compared using paired analyses. Injury data included NBA both regular season and postseason injuries. Collectively, these variables provided a comprehensive assessment of each player’s workload, performance, and injury profile, allowing for a thorough evaluation of how Olympic participation may influence subsequent NBA season outcomes.

Several variables were adjusted because of special circumstances. Due to the 2012 NBA lockout and the 2020 NBA Covid-19 season, which involved shortened schedules, percentage of games played was used to standardize comparisons across seasons, rather than to rely on raw game totals alone. For any players that were traded during the season, the player’s net rating was analyzed for the team he played the majority of the games for that season. An “injury” was considered if a player missed ≥3 games. This threshold was selected because many NBA teams are very cautious with the management of players because of the rigorous scheduling the NBA places upon them, so injuries may not be severe and can often be more related to management if a player has missed so few games. Additionally, injuries were classified by anatomic location and by type (soft tissue or fracture), and recurrence during the regular season or postseason was also recorded. Injuries in the preseason were excluded from the study. Injuries in the last 2 games of the regular season were not considered, either, as many teams rested their top players to prevent any potential injuries from occurring, especially if the team was in playoff contention.

Multiple sources were used to obtain the information, including NBA.com and Basketball-Reference as primary data sources, with supplemental verification from StatMuse and ESPN. These sources were cross-referenced throughout the data acquisition process, and injury events were confirmed across ≥2 independent sources when available to ensure accuracy and consistency.

### Statistical Analysis

Descriptive statistics were used for cohort characteristics. Paired *t* tests were performed to determine differences in game participation, minutes played, and injuries sustained in seasons immediately before and after Olympic involvement. Paired *t* tests were also utilized to compare player performancemetrics across these 2 seasons. Statistical significance was set at *P* < .05, with 95% CIs and Cohen *d* reported to characterize the precision and magnitude of observed differences. Multiple linear regression models were constructed for each outcome to account for potential confounding variables, including age, years of NBA experience, Olympic minutes played, and preceding season injury history. All analyses were performed using R (version 4.5.1; R Foundation for Statistical Computing). Given the retrospective nature of the study and inclusion of all eligible players meeting predefined criteria, a power analysis was not performed.

## Results

A total of 72 NBA players representing the United States in the Olympic Games since 2004 were identified. Five players were excluded: 2 resulting from lack of participation in an NBA season before the Olympics, 2 because of preexisting injury limiting the preceding season, and 1 with excessive non–injury related absences, resulting in a final sample of 67 players. The mean age of the players was 27.1 years, with a mean of 7 years of experience in the NBA (Table 1). Most players were either small forward (35.8%), point guards (19.4%), or shooting guards (19.4%). Olympic participation was relatively evenly distributed across the study period.

**Table 1:** Baseline Characteristics of the Study Cohort (N = 67)*^*a*^*

Age, y		27.1 ± 3.9 (20-40)
Experience, y		7.0 ± 4.0 (1-21)
Position		
	PG	13 (19.4)
	SG	13 (19.4)
	SF	24 (35.8)
	PF	12 (17.9)
	C	5 (7.5)
Olympic year, players		
	2004	11 (16.4)
	2008	12 (17.9)
	2012	11 (16.4)
	2016	11 (16.4)
	2020	10 (14.9)
	2024	12 (17.9)
Minutes played during Olympics		125 ± 50.0 (20-230)

a Data are presented as mean ± SD (range) or n (%). C, center; PF, power forward; PG, point guard; SF, small forward; SG, shooting guard.

When comparing the game participation of NBA players in the season before and after their participation in the Olympics, a significant decrease was observed in the percentage of games played in the subsequent season (89.2% ± 10.3% vs 83.2% ± 18.7%; *P* = .01; *d* = −0.31) (Table 2). There was no significant difference in total minutes played between seasons (*P* = .55; *d* = −0.07) (Table 2). Furthermore, there was a significant increase in the number of injuries experienced during the season following Olympic participation (0.7 ± 0.8 vs 0.9 ± 1.0; *P* = .04; *d* = 0.25) (Table 2).

**Table 2:** Comparison of Game Participation, Total Minutes Played, and Injury Incidence per Player in the NBA Seasons Before and After Olympic Participation*^*a*^*

	Mean ± SD	*P*	95% CI	Cohen *d*
Games in preceding season, %	89.2 ± 10.3	**.01**	–10.641 to −1.257	–0.31
Games in subsequent season, %	83.2 ± 18.7			
Total minutes in preceding season	2481.7 ± 409.5	.55	–205.239 to 109.687	–0.07
Total minutes in subsequent season	2434.0 ± 621.6			
Injuries in preceding season	0.7 ± 0.8	**.04**	0.008 to 0.529	0.25
Injuries in subsequent season	0.9 ± 1.0			

a Injury values represent the mean number of injuries per player during the respective NBA season. NBA, National Basketball Association. Statistically significant findings are shown in bold.

No differences were found in any metric of playing performance in NBA players before and after their participation in the Olympics (Table 3). Players had similar full-season plus-minus metrics (*P* = .44; *d* = 0.096) and net rating (*P* = .65; *d* = 0.055) across the preceding and subsequent seasons of Olympic participations.

**Table 3:** Comparison of Players’ Performance Metrics Before and After Olympics Participation*^*a*^*

	Mean ± SD	*P*	95% CI	Cohen *d*
Plus-minus in preceding season	224.6 ± 268.1	.44	–43.612 to 99.970	0.096
Plus-minus in subsequent season	252.8 ± 289.4			
Net rating in preceding season	4.3 ± 5.3	.65	–1.147 to 1.816	0.055
Net rating in subsequent season	4.6 ± 5.8			
Game score in preceding season	16.9 ± 3.8	.49	–0.471 to 0.981	0.086
Game score in subsequent season	17.2 ± 3.7			

a Plus-minus reflects the team point differential while the player is on the court; net rating represents the team point differential per 100 possessions while the player is on the court; and game score is a composite metric of individual player performance.

To account for potential confounding variables, multiple linear regression models were constructed for each outcome, adjusting for age, years of NBA experience, Olympic minutes played, and preceding season injury history. Preceding season values were included as baseline covariates in each respective model. Age was a significant negative predictor of both subsequent season games played (B = −4.210; *P* = .046) and total minutes played (B = −132.853; *P* = .049). Preceding injury history was the strongest predictor of subsequent season injuries (B = 0.440; *P* = .002).

Olympic minutes played emerged as a significant positive predictor of plus-minus (B = 1.585; *P* = .02), net rating (B = 0.033; *P* = .01), and game score (B = 0.015; *P* = .03) in the subsequent season. No significant associations were observed between Olympic minutes and availability outcomes. Full regression model results are presented in Table 4.

**Table 4:** Multiple Linear Regression Models Predicting Subsequent NBA Regular Season Outcomes Following Olympic Participation*^*a*^*

Predictor	B	*P*	95% CI
% Games played: *R*^2^ = 0.155; adj *R*^2^ = 0.085			
Preceding games played, %	−0.068	.83	−0.703 to 0.568
Age, y	−4.210	**.046**	−8.333 to −0.087
Years of experience	3.475	.09	−0.565 to 7.516
Olympic minutes	0.047	.31	−0.045 to 0.138
Preceding injuries	−5.716	.15	−13.462 to 2.031
Total minutes: *R*^2^ = 0.183; adj *R*^2^ = 0.116			
Preceding total minutes	0.163	.45	−0.262 to 0.589
Age, y	−132.853	**.049**	−265.637 to −0.069
Years of experience	100.228	.13	−30.258 to 230.713
Olympic minutes	2.326	.15	−0.824 to 5.476
Preceding injuries	−82.589	.41	−282.836 to 117.658
Injuries: *R*^2^ = 0.300; adj *R*^2^ = 0.255			
Preceding injuries	0.440	**.002**	0.173 to 0.707
Age, y	0.009	.93	−0.190 to 0.209
Years of experience	0.095	.34	−0.102 to 0.292
Olympic minutes	−0.004	.06	−0.009 to 0.000
Plus-minus: *R*^2^ = 0.316; adj *R*^2^ = 0.260			
Preceding plus-minus	0.420	**.001**	0.180 to 0.660
Age, y	−14.204	.62	−70.333 to 41.925
Years of experience	−0.895	.97	−56.300 to 54.511
Olympic minutes	1.585	**.02**	0.307 to 2.862
Preceding injuries	−50.691	.20	−129.041 to 27.660
Net rating: *R*^2^ = 0.28; adj *R*^2^ = 0.226			
Preceding net rating	0.383	**.003**	0.140 to 0.626
Age, y	−0.303	.60	−1.447 to 0.842
Years of experience	0.069	.90	−1.061 to 1.200
Olympic minutes	0.033	**.01**	0.007 to 0.059
Preceding injuries	−1.193	.13	−2.763 to 0.377
Game score: *R*^2^ = 0.561; adj *R*^2^ = 0.525			
Preceding game score	0.571	<**.001**	0.379 to 0.763
Age, y	−0.566	.06	−1.151 to 0.019
Years of experience	0.374	.21	−0.210 to 0.958
Olympic minutes	0.015	**.03**	0.002 to 0.029
Preceding injuries	0.306	.45	−0.501 to 1.113

a Data are presented as unstandardized regression coefficients (B) with corresponding *P* values and 95% CIs. NBA, National Basketball Association. Statistically significant values (*P* < .05) are shown in bold.

The distribution of games missed by injury location during the post-Olympic season is shown in Table 5; percentages reflect the proportion of the total games missed (N = 807).

**Table 5:** Distribution of Games Missed by Injury Location Post-Olympic Season (N = 807 Total Games Missed)*^*a*^*

Anatomic Region	Specific Anatomic Site	Games Missed, n (%)
Lower extremity	Knee	181 (22.4)
	Ankle	92 (11.4)
	Foot	46 (5.7)
	Quadriceps	44 (5.5)
	Achilles	31 (3.8)
	Hamstring	20 (2.5)
	Adductor	18 (2.2)
	Calf	17 (2.1)
	Groin	16 (2.0)
	Heel	3 (0.4)
	Hip	3 (0.4)
Upper extremity	Wrist	73 (9.0)
	Hand	72 (8.9)
	Thumb	46 (5.7)
	Shoulder	31 (3.8)
	Finger	4 (0.5)
	Elbow	3 (0.4)
Core/trunk	Abdomen	59 (7.3)
	Back	32 (4.0)
	Neck	16 (2.0)

a Injuries were defined as absences of ≥3 games during the post-Olympic season.

## Discussion

This study examined whether participation in the Olympic Games is associated with subsequent NBA availability, injury incidence, and on-court performance. Our analysis demonstrated a statistically significant reduction in the percentage of games played in the season following Olympic participation compared with the preceding season (89.2% ± 10.3% vs 83.2% ± 18.7%; *P* = .01), accompanied by a significant increase in injury incidence (0.7 ± 0.8 vs 0.9 ± 1.0 injuries; *P* = .04). In contrast, no significant differences were observed in key performance metrics, including plus-minus, net rating, and game score. Importantly, despite the reduction in games played, no significant difference was observed in total minutes played between seasons, indicating that players maintained comparable workloads and underscoring a distinction between player availability and overall playing time. These findings suggest that while Olympic participation may be associated with increased subsequent injury risk and reduced availability, it does not meaningfully alter overall NBA performance.

The observed reduction in games played and increase in injuries following Olympic participation may reflect cumulative fatigue and delayed recovery resulting from a compressed annual competitive schedule. It is also possible that missed games reflect intentional rest or load management decisions, rather than true injuries, which may limit interpretation of the observed association. NBA players who participate in the Olympic Games often have only 6 to 8 weeks of rest within 52 weeks, substantially limiting opportunities for physiologic recovery and reconditioning. Given this rigorous schedule, athletes are exposed to increased cumulative physical strain, which may heighten susceptibility to injury, particularly soft tissue injuries. This interpretation is consistent with prior literature demonstratingassociations between increased fatigue, elevated game load, and higher injury risk in professional basketball players.^2,5,7,20,24^ These relationships have been observed across workload metrics such as minutes played and recovery time, with higher playing time associated with increased injury risk and greater rest associated with reduced risk in the NBA.^20^ Studies in other high-intensity, intermittent sports such as rugby and soccer have identified workload accumulation and fatigue as key contributors to soft tissue injury risk.^10,13,15,23^ While basketball differs from other team sports, findings from rugby and soccer may provide relevant context, as these sports also involve intermittent high-intensity demands and periods of constrained recovery that may contribute to injury risk. In our cohort, 95.2% of injuries sustained in the subsequent season were soft tissue injuries, supporting the hypothesis that workload-related strain, rather than isolated traumatic events, may be associated with the observed increase in injury incidence. From a clinical and performance perspective, these findings have important implications for sports medicine and performance staff. They underscore the importance of individualized workloadmonitoring, targeted recovery strategies, and early-season load management for players returning from international competition, which may support efforts to mitigate injury risk.

Other factors that may contribute to increased injury risk include the travel demands experienced by NBA players. During the NBA season, teams travel extensively across the United States and Canada, often with frequent changes in time zones and limited recovery between games. In contrast, Olympic-related travel is typically front-loaded, involving international travel to the host country followed by relatively limited travel and more stable time zone exposure during the tournament itself. Prior research in soccer has demonstrated that players who sustained injuries accumulated greater international travel distances, flight hours, and time zones crossed compared with noninjured controls, suggesting that travel may act as a contributing risk factor.^9^ Although direct literature examining travel-related injury risk in NBA players is limited,existing evidence indicates that air travel can adversely affect sleep, circadian alignment, recovery, andperformance and should be considered an additional workload-related stressor in professional basketball.^8,16,19,28^ While the travel patterns differ between the NBA season and Olympic competition, factors such as long-distance travel, potential circadian disruption at the onset of Olympic participation, and the physical demands of competition may be associated with the increased injury risk observed in our cohort.

Supplementary regression analyses further contextualized these findings by identifying age and preceding injury history as significant contributing factors to subsequent season outcomes. Age was a significant negative predictor of both games played (*P* = .046) and total minutes played (*P* = .049) in the subsequent season, suggesting that older players may be less resilient to the cumulative demands of an Olympic year and may require more deliberate load management strategies upon returning to NBA competition. Preceding injury history was the strongest predictor of subsequent season injuries (*P* = .002), indicating that players who entered the Olympic year with an existing injury burden carried a meaningfully higher risk of sustaining injuries in the following season. Such findings emphasize the importance of pre-Olympic injury screening and individualized return-to-play planning for players with prior injury history. Of note, Olympic minutes played emerged as a significant positive predictor of plus-minus (*P* = .02), net rating (*P* = .01), and game score (*P* = .03) in the subsequent season. Rather than suggesting a performance benefit of Olympic participation, this finding may reflect a selection effect, whereby higher-caliber players who received greater Olympic playing time were inherently more likely to sustain higher performance regardless of their international participation. This interpretation is consistent with the absence of significant differences in key performance metrics observed in the primary analysis and is further supported by literature in professional hockey reporting similarly minimal post-Olympic performance effects despite increased competitive exposure, suggesting that any theoretical benefits of enhanced conditioning are likely offset by accumulated fatigue.^4,6,21^ Although derived from a different sport, these findings remain relevant given the shared demands of elite international competition and condensed recovery periods, supporting the absence of a measurable post-Olympic performance effect observed in our cohort. These findings reinforce the primary results while highlighting the role of individual player characteristics in moderating the effects of Olympic participation on subsequent NBA outcomes.

Limited research has examined the effects of Olympic participation on athletes across different sports, making direct comparisons challenging. Exploratory reports in soccer have suggested increases in both injury incidence and injury severity following major international tournaments, including higher rates of lower extremity injuries.^25^ In our cohort, lower extremity injuries involving the knee and ankle were the most prevalent following Olympic participation, representing a potential area of overlap with findings reported in other sports. Nevertheless, these comparisons should be interpreted cautiously, and further sport-specific investigation is warranted.

### Limitations

Several limitations should be considered when interpreting these findings. Injury severity was assessed using a threshold of ≥3 missed games, selected to better capture clinically meaningful absences reflective of cumulative workload-related strain in the context of the NBA’s tightly congested schedule. However, this definition introduces the potential for misclassification, as some absences of ≥3 games may still reflect precautionary management decisions, while minor injuries resulting in shorter absences may not be captured. The use of publicly available data further limited the ability to distinguish between acute and chronic injuries, and injury identification relied on publicly available reports that may be subject to variability in reporting accuracy and lack detailed clinical verification, although injuries were cross-checked across ≥2 independent sources when available. Playoff performance could not be evaluated, as 28 players did not participate in ≥1 of the playoff periods before or after Olympic participation, and performance metrics such as game score, plus-minus, and net rating demonstrated considerable variability influenced by opponent strength and series dynamics, making it difficult to attribute changes specifically to Olympic participation. A matched control group was not feasible because of substantial heterogeneity in player workload, utilization, and career trajectories, as well as the fact that Olympic participation represents a unique exposure not readily replicated by other competitions or training environments; instead, each player’s preceding season was used as an internal control, though this approach may introduce bias related to temporal changes including aging, evolving player roles, team context, and cumulative workload. Preseason data were not collected, as NBA players generally have limited preseason participation regardless of Olympic involvement, and the inclusion of only US Olympic participants may limit generalizability to international NBA players, who may differ in role, utilization patterns, and workload exposure. Accordingly, all findings should be interpreted as associative rather than causal and considered within a hypothesis-generating framework, with future studies utilizing larger, more diverse cohorts needed to further characterize the relationship between Olympic participation and subsequent NBA outcomes.

## Conclusion

Olympic participation was associated with decreased player availability and increased injury incidence in the subsequent NBA season compared with the preceding season, without meaningful changes in performance metrics or total minutes played. Supplementary regression analyses identified age and preceding injury history as significant contributors to subsequent season outcomes, suggesting that older players and those with existing injury burdens may be particularly vulnerable following Olympic participation. These findings collectively suggest that the compressed competitive schedule associated with Olympic involvement, combined with limited offseason recovery, may be associated with overuse-related injury and reduced player durability in the subsequent NBA season. From a clinical perspective, these results underscore the importance of individualized workload monitoring, targeted recovery strategies, and proactive injury screening for elite basketball players returning from international competition, with the goal of supporting long-term athlete health and sustained performance.
